# The addition of whole soy flour to cafeteria diet reduces metabolic risk markers in wistar rats

**DOI:** 10.1186/1476-511X-12-145

**Published:** 2013-10-11

**Authors:** Gláucia Ferreira Andrade, Crislaine das Graças de Almeida, Ana Cristina Rocha Espeschit, Maria Inês de Souza Dantas, Laércio dos Anjos Benjamin, Sonia Machado Rocha Ribeiro, Hércia Stampini Duarte Martino

**Affiliations:** 1 Department of Nutrition and Health, CCB-II, Federal University of Viçosa, Viçosa, Minas Gerais, Brazil; 2 Department of Veterinary Medicine, Federal University of Viçosa, Viçosa, Minas Gerais, Brazil

**Keywords:** Soybean, Cafeteria diet, Functional foods, Intestinal histomorphometry, Lipid peroxidation

## Abstract

**Background:**

Soybean is termed a functional food because it contains bioactive compounds. However, its effects are not well known under unbalanced diet conditions. This work is aimed at evaluating the effect of adding whole soy flour to a cafeteria diet on intestinal histomorphometry, metabolic risk and toxicity markers in rats.

**Methods:**

In this study, 30 male adult Wistar rats were used, distributed among three groups (n = 10): AIN-93 M diet, cafeteria diet (CAF) and cafeteria diet with soy flour (CAFS), for 56 days. The following parameters were measured: food intake; weight gain; serum concentrations of triglycerides, total cholesterol, HDL-c, glycated hemoglobin (HbA1c), aspartate (AST) and alanine (ALT) aminotransferases and Thiobarbituric Acid Reactive Substances (TBARS); humidity and lipid fecal content; weight and fat of the liver. The villous height, the crypt depth and the thickness of the duodenal and ileal circular and longitudinal muscle layers of the animals were also measured.

**Results:**

There was a significant reduction in the food intake in the CAF group. The CAFS showed lower serum concentrations of triglycerides and serum TBARS and a lower percentage of hepatic fat, with a corresponding increase in thickness of the intestinal muscle layers. In the CAF group, an increase in the HbA1c, ALT, lipid excretion, liver TBARS and crypt depth, was observed associated with lower HDL-c and villous height. The addition of soy did not promote any change in these parameters.

**Conclusions:**

The inclusion of whole soy flour in a high-fat diet may be helpful in reducing some markers of metabolic risk; however, more studies are required to clarify its effects on unbalanced diets.

## Abstract (pt)

**Resumo:**

**Introdução**

A soja é um alimento funcional por conter compostos bioativos. Entretanto, seu efeito não é bem conhecido em situações de desequilíbrio alimentar. Este trabalho objetivou avaliar o efeito da adição de farinha integral de soja em uma dieta de cafeteria sobre a histomorfometria intestinal, risco metabólico e marcadores de toxicidade em ratos.

**Métodos**

Trinta ratos machos adultos Wistar foram utilizados no estudo, os quais foram distribuídos em três grupos (n = 10): dieta AIN-93 M, dieta de cafeteria (CAF) e dieta de cafeteria com adição de farinha de soja (CAFS), por 56 dias. Foram mensurados o consumo alimentar, ganho de peso, concentrações séricas de triacilgliceróis, colesterol total, HDL-c, hemoglobina glicada, enzimas aspartato (AST) e alanina (ALT) aminotransferases e TBARS; umidade e conteúdo de lipídio das fezes; peso e gordura hepática. A altura de vilosidade, profundidade de cripta e espessura das camadas musculares circular e longitudinal do duodeno e íleo dos animais foram também mensurados.

**Resultados**

Houve redução significativa do consumo alimentar no grupo CAF. CAFS apresentou menores concentrações séricas de triacilgliceróis e TBARS e menor percentual de gordura hepática; e maior espessura das camadas musculares. No grupo CAF foi observado aumento da HbA1c, ALT, excreção lipídica, TBARS hepático e profundidade de cripta, associado a menor HDL-c e altura de vilosidade. A adição de farinha integral de soja não acarretou mudança desses parâmetros.

**Conclusões**

A inclusão de farinha integral de soja em uma dieta hiperlipídica pode auxiliar na redução de alguns marcadores de risco metabólico, entretanto, mais estudos são necessários para esclarecer seu efeito em dietas desbalanceadas.

## Background

Obesity has become a major public health problem worldwide. It is followed by a significantly higher risk of developing chronic diseases such as type 2 diabetes, hypertension, dyslipidemia and atherosclerosis. The prevalence of these diseases, commonly related to Metabolic Syndrome, has overtaken malnutrition and infectious diseases, contributing to morbidity, across the world [1]. The rapid increase in obesity is found to be linked more to lifestyle changes in the population, produced by the consumption of hypercaloric diets, rich in fats and simple sugars, and a sedentary lifestyle, than to genetic factors [2]. The cafeteria diet is an experimental model used to study the effects of Western dietary pattern on animal health, because it is able to represent the food intake of modern societies, characterized by meals prepared at cafeterias and Fast Foods joints [3,4]. The cafeteria diet is a robust experimental model of human metabolic syndrome, capable of inducing obesity, glucose intolerance and inflammation in rats [4].

Once the origin of obesity and metabolic disorders is established, often the correct diet and nutrition may also form part of the solution, providing health benefits. Currently, the interest evinced by the population for dietary interventions to control excess body weight, dyslipidemia and hyperglycemia has increased. Soy has been widely studied because it contains vegetable protein of high biological value, bioactive peptides, polyunsaturated fatty acids and dietary fiber [5]. It also possesses phytochemicals, including isoflavones and phytate, which may contribute to the reduction of the risk factors of cardiovascular disease (CVD), type 2 diabetes mellitus and atherosclerosis [6-8]. However, it has been reported that soy, in addition to a balanced diet, changes the intestinal morphology, impairing the nutrient absorption in the small intestine [9,10].

Several studies have shown that soy protein in its isolated or textured form has lipid-lowering properties, reducing the serum levels of total cholesterol, LDL cholesterol, VLDL and triglycerides, and decreasing the susceptibility of lipoproteins to oxidation, which represents a risk factor related to the early development of atherosclerosis. The mechanisms that promote the lipid-lowering effect of soy protein have not yet been fully understood; however, they may be related to the inhibition of dietary cholesterol absorption, enhanced excretion of bile acids and an increased number of tissue activity receptors of LDL-c [7,11-13].

Some evidences of the direct action of the peptide or protein, rather than the other chemical components present in soybean were revealed [14,15]. Others showed that the cholesterol-lowering effect of soy protein was associated with the presence of isoflavones [7,16], which also reveal an antioxidant action, helping to reduce the lipid peroxidation [17-20].

Therefore, the effects of soy protein and isoflavones have been isolated, investigated or linked together, which often do not reveal the reality of the full consumption of the soy, because the soy also contains soy fiber and polyunsaturated fatty acids, phytates, protease inhibitors, lipoxygenase and oligosaccharides, which have both functional and antinutritional properties.

The soybean cultivar UFVTN 105AP for human consumption offers high protein concentration and in order to improve the nutritional and sensory characteristics of soybean is free of lipoxygenase [21]. The development of the soybean culture is accompanied by the need to evaluate the beneficial or adverse effects of the soy on the body, associated with a cafeteria diet, the main characteristic of modern lifestyle.

The objective of this work was to evaluate the effect of adding soy flour to the cafeteria diet on the lipid and carbohydrate metabolism, lipid peroxidation, in the serum concentration of the enzymes alanine aminotransferase and aspartate, and in the intestinal and liver histomorphometry of adult Wistar rats.

## Results

The weight gained by the animals did not differ between the groups; however, the total food consumption of the animals was lower (p < 0.05) in the groups fed on the cafeteria diet, CAF (+) and CAFS compared with the negative control group, AIN-93 M (-). The animals in the CAF (+) and CAFS groups reduced their food intake without any alteration in weight gain. This fact promoted the increase (p < 0.05) of Feed Efficiency Ratio (FER) (Table 1). The serum levels of the total cholesterol and the total HDL-c/cholesterol ratio did not differ (p ≥ 0.05) among the experimental groups. The serum triglyceride levels did not differ between the groups CAF controls (+) and AIN-93 M (-), although they were lower (p <0.05) in CAFS. The CAF (+) and CAFS diets reduced the serum HDL-c (p < 0.05) and increased the serum concentration of the glycated hemoglobin (p < 0.05) compared with the group AIN-93 M (-). The animals in the CAF (+) and CAFS groups excreted greater amounts of lipids (p < 0.05) in the feces compared with those fed with on the AIN-93 M (-) diet (Table 1).

**Table 1:** Metabolic parameters of animals treated with different diets after 56 days of experiment

Parameters*	**Experimental groups**		
	**AIN – 93 M**	**CAF**	**CAFS**
Weigth gain (g)	151.00^a^ ± 31.75	170.40^a^ ± 53.80	162.10^a^ ± 29.98
DI (g)	1786.56^a^ ± 45.46	1402.94^b^ ± 51.61	1415.46^b^ ± 77.24
FER	0.08^a^ ± 0.017	0.11^b^ ± 0.033	0.114^b^ ± 0.020
Energy intake (Kcal/dia)	81.34	94.73	96.31
Triglycerides (mg/dL)	215.22 ^a^ ± 40.74	215.10^a^ ± 47.07	163.32^b^ ± 58.18
Total cholesterol (mg/dL)	128.21^a^ ± 24.48	105.13^a^ ± 10.97	115.65^a^ ± 22.77
HDL (mg/dL)	47.43^a^ ± 8.35	38.18^b^ ± 6.74	37.87^b^ ± 7.20
HDL/total cholesterol	0.37^a^ ± 0.05	0.35^a^ ± 0.05	0.33^a^ ± 0.07
HbA1c (%)	9.49^a^ ± 2.58	16.53^b^ ± 5.98	15.69^b^ ± 7.48
Fecal humidty (%)	27.96^a^ ± 7.22	22.09^b^ ± 7.98	24.11^ab^ ± 3.68
Fecal lipid (%)	3.10^a^ ± 0.55	13.29^b^ ± 1.56	13.65 ^b^ ± 1.39
Liver weight (g)	15.95^a^ ± 2.36	16.58^a^ ± 2.18	15.44^a^ ± 2.13
HIS	3.45^a^ ± 0.35	3.44^a^ ± 0.37	3.24^a^ ± 0.21
Liver fat (%)	0.61^a^ (0.06-10.54)	0.07^a^ (0.01-15.29)	0.17^b^ (0.01-3.84)
ALT (UI/L)	90.03^a^ ± 12.00	130.76^b^ ± 22.03	127.85^b^ ± 25.33
AST (UI/L)	338.59^a^ ± 87.93	380.11^a^ ± 113.07	422.35^a^ ± 163.13

Results are expressed as mean ± SD for all parameters with exception of liver fat witch is represented by median (maximum and minimum). AIN-93 M diet: negative control group (n = 10/group); cafeteria diet (CAF): positive control group (n = 10/group); and caferia diet with whole soybean flour (CAFS) (n = 10/group). To compare the experimental groups with exception of LH Duncan test was used. To compare LF parameter between experimental groups Kruskall-Wallis test was used. For each evaluated characteristic means followed by the same letter in the lines are not significantly different at 5% probability.

*ALT, enzyme alanine aminotransferase; AST, enzyme aspartate aminotransferase; DI, dietary intake; FER, feed efficiency ratio; HbA1c, glycated hemoglobin; HDL, high density lipoprotein; HIS, hepatosomatic index.

The hepatosomatic index did not differ (p > 0.05) among the experimental groups. But it was observed that the CAFS group has less accumulated fat in the liver (p < 0.05) (Table 2). The serum levels of the enzyme aspartate aminotransferase did not differ (p ≥ 0.05) among the experimental groups, although an increase in the serum concentrations of the enzyme alanine aminotransferase (p < 0.05) was observed with CAF (+) and CAFS (Table 1).

The serum TBARS levels were lower (p < 0.05) with CAF (+) than with other diets. However, the TBARS in the liver and testicles were higher (p < 0.05) with CAF (+) and the CAFS diet compared with the AIN-93 M (-). The CAFS diet increased the TBARS concentration in the lung (p < 0.05). However, in the kidney, the concentration of the TBARS was found to be significantly higher (p < 0.05) in the CAF (+) group (Table 2).

**Table 2:** TBARS* concentrations in different organs of animals fed with experimental diets

**Organs**	**Experimental groups**		
	**AIN 93-M**	**CAF**	**CAFS**
Serum	1.64^a^ ± 0.47	1.23^b^ ± 0.23	1.74^a^ ± 0.16
Liver	0.09^b^ ± 0.03	0.40^a^ ± 0.10	0.44^a^ ± 0.152
Lung	77.43^b^ ± 14.00	76.54^b^ ± 45.28	235.46^a^ ± 77.99
Testicle	10.13^b^ ± 3.69	17.94^a^ ± 9.50	18.07^a^ ± 7.82
Kidney	92.30^b^ ± 15.59	120.07^a^ ± 25.64	97.82^b^ ± 19.13

Results are expressed as mean ± SD. AIN-93 M diet. Negative control group (n = 10/group); cafeteria diet (CAF), positive control group (n = 10/group); and caferia diet with whole soybean flour (CAFS) (n = 10/group). To compare the experimental groups Duncan test was used. For each evaluated characteristic means followed by the same letter in the lines are not significantly different at 5% probability.

*TBARS, test thiobarbituric acid reactive substances.

The animals in the CAF (+) and CAFS groups showed a reduction in villous height (p <0.05) and an increase in the duodenal crypt depth (p < 0.05). The thicknesses of the circular and longitudinal muscles was greater (p < 0.05) in the CAFS group (Table 3). In the ileal portion of the small intestine the villous height decreased (p < 0.05) in those rats on the CAFS diet compared with the negative control (AIN-93 M) and increased (p < 0.05) compared with the positive control, CAF. The measurements of crypt depth and layer thickness of the longitudinal muscle were greater (p <0.05) in the CAFS group. The thickness of the circular muscle layer in the CAF (+) and CAFS groups was similar (p > 0.05), but higher (p < 0.05) when compared with the negative control, AIN-93 M (Table 3).

**Table 3:** Measurements (mm) of duodenum and ileum of animals

**Portion of small intestine**	Parameters*	**Experimental groups**		
		**AIN-93 M**	**CAF**	**CAFS**
Duodenum	VH	157.1^a^ ± 22.4	123.4^b^ ± 33.4	130.2^b^ ± 18.5
	CD	223.6^b^ ± 24.2	258.1^a^ ± 47.7	262.6^a^ ± 34.2
	TLML	63.0^b^ ± 19.2	63.1^b^ ± 18.7	89.9^a^ ± 30.4
	TCML	88.1^b^ ± 32.4	79.0^b^ ± 17.5	108.0^a^ ± 31.4
Ileum	VH	598.2^a^ ± 80.8	426.5^c^ ± 122.1	503.9^b^ ± 79.9
	CD	235.6^b^ ± 35.7	243.3^b^ ± 42.8	272.9^a^ ± 58.2
	TLML	69.9^b^ ± 16.8	76.8^b^ ± 11.5	87.9^a^ ± 15.0
	TCML	50.8^b^ ± 12.6	57.4^a^ ± 12.2	59.1^a^ ± 22.8

Results are expressed as mean ± SD. AIN-93 M diet: negative control group (n = 10/group); cafeteria diet (CAF): positive control group (n = 10/group); and cafeteria diet with whole soybean flour (CAFS) (n = 10/group). To compare the experimental groups Duncan test was used. For each evaluated characteristic means followed by the same letter in the lines are not significantly different at 5% probability.

*CD, crypt depth; TCML, thicknesses of the circular muscle layer; TLML, thicknesses of the longitudinal muscle layer; VH, villus height.

## Discussion

The cafeteria diets, with or without the addition of whole soy flour, despite being hyperlipidemic and hypercaloric, did not affect weight gain in the animals. Therefore, weight gain is an indirect parameter to the evaluated inflammatory state. It is known that the cafeteria diet is considered a robust model of the human metabolic syndrome with reference to the liver and adipose tissues [4]. There is evidence that the diet-induced metabolic dysfunctions observed in the rats are independent of the degree of increase in total body weight [22]. The low food intake in the CAF group was probably due to their higher energy density. It is known that rats tend to maintain their energy intake at a relatively fixed rate [23,24]. This result is in accordance with the higher FER in the CAF and CAFS groups. Rats from the CAF group required less food intake to gain one gram of weight. As CAFS do not differ from the CAF, it could be that the fiber content was insufficient to reduce the food intake.

The serum concentrations of total cholesterol, triglycerides and hepatic fat content were not changed with the consumption of the CAF diet. These changes might have occurred because the rats increased the excretion of fecal lipids, by approximately four times. The addition of whole soy flour in the CAF group did not change these parameters; however, it decreased (p < 0.05) the serum triglycerides. This effect of whole soy flour of lowering the triglyceride level can be attributed to the soy protein and or the presence of isoflavones and dietary fiber [18,25]. The hypotriglyceride property of soybean was observed in other studies as well, with animals and humans on balanced diets [25-29]. It was interesting to note that whole soy flour in the cafeteria diet decreased the liver fat (p < 0.05), countering the effect of the high-fat diet. This fact shows the biologic protective effect of soy flour in response to metabolic syndrome, as an effective prevention of non-alcoholic fatty liver disease (NFLD). It was observed that the CAF diet increased the serum alanine aminotransferase enzyme (ALT) levels in the liver of the animals. This concurs with the results of MacQueen et al. [30], in animals treated with the cafeteria diet. The liver injury could have been caused by steatosis, a disease state characterized by inflammation, fibrosis, cell death and insulin resistance [31]. The addition of whole soy flour to the cafeteria diet did not result in the ALT concentration returning to levels similar to those of the negative control, although it decreased the hepatic fat.

A reduction in the serum HDL-C concentration was noted in the group receiving the cafeteria diet, positive control, similar to that the results found by MacQueen et al. [30], in Sprague Dawley rats. Due to the high HDL-c levels acting as a protective factor against possible cardiovascular events [32], the cafeteria diet had adverse health effects, which were not attenuated by the presence of the soy flour.

The animals treated with the high-fat diet cafeteria CAF showed an increase in the HbA1c. This kind of diet can affect the insulin-mediated glucose metabolism [30,33,34], by decreasing the glucose transporter 4 expression [35], changing via insulin-signaling [36] in the muscle and adipose tissues. Despite being reported in the literature that soy consumption has a positive effect on the glucose metabolism [18], this relationship was not observed in the present study when whole soy flour was added to an unbalanced diet. The whole soy flour was the intake at the dietetic level by the rats, and later it could have been difficult to counter the effect of the high-fat diet.

The CAF diet decreased the serum lipid peroxidation. It is likely that the animals treated with the cafeteria diet have higher circulating levels of free saturated fatty acids. It is known that the saturated fatty acids are more resistant to peroxidation than the unsaturated fatty acids, in rats on the AIN-93 M (-) diet [32,37]. Also, soybean flour has a high concentration of polyunsaturated fatty acids [5] which can undergo peroxidation. Therefore, when whole soy flour was added to the CAF diet, it increased the lipid peroxidation to the same level (p > 0.05) as the negative control, returning at physiological levels. This can be attributed to the antioxidant compounds present in soy flour, such as isoflavones [38-40] and vitamin E [41], which may protect it against lipid peroxidation [42].

In the tissues, the CAF diet increased the lipid peroxidation in the liver, testicles and kidney. When whole soy flour was added to the CAF diet, this parameter did not change (p > 0.05) either in the liver or testicles, although it decreased in the kidney (p < 0.05). This fact demonstrated that bioactive compounds have different bio-accessibility in the biological tissues. In the lung, the CAFS diet showed lipid peroxidation approximately three times higher than the CAF and AIN-93 M (-) diets. Bioactive compounds of whole soy flour can perform the redox cycle with oxygen and become pro-oxidant in the lung, which has a high oxygen concentration. There are strong suggestions that dietary antioxidant can induce reactive oxygen species and thereby trigger an adaptive stress response and hormesis, and as a result the cells will increase their production of cytoprotection against oxidative stress [43].

Regarding intestinal morphology, it was observed that the cafeteria diet reduced the villous height and increased the crypt depth of the duodenum, which could adversely impair nutrient absorption. However, these results differ from those obtained by Scoaris et al. [2], who demonstrated an increase in the villous height and crypt depth of the jejunum of the rats fed on the cafeteria diet. The CAF diet effect is expected, because of its inflammatory potential, which has been reported in the literature [44], to significantly compromise the integrity of the intestinal mucosa as a whole. However, the relationship between a high-fat diet, imbalanced gut microbiota and the host pathophysiology remains to be elucidated. It was discovered that bile acid, the main component of bile, is a host factor that regulates the composition of the cecal microbiota in rats. It is because bile secretion increases on the high-fat diet, and bile acids generally exhibit strong antimicrobial activity, it was speculated that the bile acids would be a determinant of the gut microbiota in response to a high-fat diet [45]. The changes observed in the rat cecal microbiota triggered by the administration of cholic acid (the most abundant bile acid in human biliary bile) resemble those found in the animals fed on high-fat diets.

The effect of adding whole soy flour to the CAF diet caused no change in the villi and crypts, but increased the thicknesses of the circular and longitudinal muscle layers. Then, soybean contributed to the increase in the intestinal muscle layers, which led to greater intestinal peristalsis. Perhaps, because of the presence of the soluble fiber and soy oligosaccharides, besides the allergenic factors, such as protein fractions glycinin and β-conglycinin, the addition of the soy flour to the cafeteria diet could not reverse the malefic effect of the cafeteria diet on the duodenal villi [46-48]. Another component of the soybean that could exert a negative effect on the small intestine is lectin. However, in this study, the lectin had not compromised the intestinal parameters in the soybean meal diet due to the heat treatment applied to the soybean (150 °C/30 min) when preparing the meal. According to Machado et al. [49], the thermal treatments in the autoclave at 120°C for 5, 10, 15 and 25 minutes were effective in inactivating the protease inhibitors and lectins.

However, the presence of the soy flour contributed to a lower degree of villous atrophy in the ileum in relation to the cafeteria diet group, positive control, and a greater crypt depth when compared with the other groups. The villous atrophy is caused by increased cell loss by desquamation or by the lowered cell renewal process. The villous atrophy that occurs via increased cell loss is associated with the increased production of cells in the crypt, and therefore, an increase in crypt depth [50], as observed in the duodenum and ileum of animals fed on the cafeteria diet containing whole soybean flour.

## Conclusion

The present study reports that the addition of soy flour was beneficial, considering that there was a reduction in the serum triglycerides, the percentage of fat in the liver regions and an increased thickness of the intestinal muscle layers, improving gut peristalsis. However, the incorporation of a functional food such as soy in the cafeteria diet was insufficient to undo the harmful effects that this type of diet causes to health.

## Methods

### Soybean flour

The cultivar UFVTN 105AP for human consumption was provided by the Institute of Biotechnology Applied to Livestock (BIOAGRO), Federal University of Viçosa, Viçosa, MG, Brazil. It is a cultivar developed without LOX1, LOX2 and LOX3 lipoxygenases, called triple null, with a high protein content [21]. To prepare the whole soybean flour, soybeans were selected and subjected to the process of flushing with water and dried at an ambient temperature. They were then subjected to 150°C temperature for 30 minutes in a stove with air circulation, chilled and stored in polyethylene bags. The grains along with the shells were milled in knife mills and passed through a sieve of 60 (0.25 mm) mesh [21].

### Experimental animals

A total of 30 male rats were used (*Rattus norvegicus albinos, Mammalia, Wistar lineage*), from the Central Biotherium of the Center for Biological Sciences and Health, Federal University of Viçosa. The animals were purchased early, weaned and maintained in individual cages in an acclimatized environment, receiving commercial chow until they reach the adult stage (10 weeks old, ~ 300 g).

During the experimental phase, the animals were maintained in individual cages in a temperature-controlled environment of 22 ± 2°C, 12 h photoperiod, receiving their daily diet and distilled water *ad libitum*. They were divided into three groups of 10 animals each, and given experimental diets for a period of 56 days, as shown in Table 4.

**Table 4:** Experimental diets composition (g/100 g)

**Ingredients**	**AIN 93-M**	**CAF**	**CAFS**
Case in (% protein)	15.40	0	0
Saccharose	10.00	0	0
Corn starch	43.92	0	0
Dextrinized starch	15.50	0	0
Soybean oil	5.25	0	0
Microcrystalline cellulose	5.00	0	0
Mineral mix AIN93M	3.50	0	0
Vitamin mix AIN93M	1.00	0	0
L-cystine	0.18	0	0
Choline bitartrate	0.25	0	0
Whole soybean flour	0	0	27.67
Chicken liver patê	0	14.28	10.33
Sweet biscuit (Aymore®)	0	14.28	10.33
Potato Chips (Quezinha®)	0	14.28	10.33
Chocolate (Garoto®)	0	14.28	10.33
Bacon	0	14.28	10.33
Commercial chow	0	28.57	20.67

Results are expressed as mean. AIN-93 M diet, negative control group (n = 10/group); cafeteria diet (CAF), positive control group (n = 10/group); and cafeteria diet with whole soybean flour (CAFS) (n = 10/group).

### Experimental design

A 56-day controlled experimental study was conducted, for parameter analysis *in vivo* and *ex vivo.* The effects of the soy flour intake to modulate the metabolic and morphological parameters of the animals were evaluated in all three groups of animals who received the following diet pellets: i) AIN-93 M (-) [51], considered the negative control, ii) CAF (+): positive control cafeteria diet, iii) CAFS: cafeteria diet + 27.67 g of whole soy flour, which is equivalent to the amount of protein provided by AIN-93 M diet, negative control.

The cafeteria diet consisted of chicken liver pate, sweet biscuit, potato chips, milk chocolate, bacon and commercial chow in the ratio of 1:1:1:1:1:2 [3]. The caloric density of the AIN-93 M, CAF and CAFS diets was 2.54 kcal/g, 4.23 kcal/g and 4.09 kcal/g, respectively; whereas the percentage contribution of the macronutrients in the energy content of the AIN-93 M, CAF and CAFS diets were, 7.9, 58.5 and 54.2%, respectively, of energy as fat, 14.9, 12.9 and 20.7%, respectively, as protein, and 77.1, 28.6 and 25.1%, respectively, as carbohydrate. The CAF and CAFS diets were hypercaloric and hyperlipidic compared with the AIN-93 M diet (Table 5).

**Table 5:** Moisture, protein, fat, carbohydrate, ash, dietary fiber and energy density from the diets

**Nutritional values**	**AIN-93 M**	**CAF**	**CAFS**
Moisture (%)	32.20^a^ ± 0.08	23.25^b^ ± 0.45	19.80^c^ ± 0.02
Protein (g/100 g)	9.52^c^ ± 0.52	13.52^b^ ± 0.74	21.28^a^ ± 0.05
Fat (g/100 g)	2.25^c^ ± 0.65	27.19^a^ ± 0.28	24.74^b^ ± 0.07
Carbohydrate (g/100 g)	49.15^a^ ± 1.16	29.88^b^ ± 0.74	25.82^c^ ± 0.06
Ash (g/100 g)	1.87^c^ ± 0.02	3.13^b^ ± 0.14	3.67^a^ ± 0.07
Dietary Fiber (g/100 g)	5.00^a^ ± 0.13	3.02^c^ ± 0.03	4.69^b^ ± 0.02
Energy density (kcal/g)	2.55^c^ ± 0.03	4.18^a^ ± 0.01	4.11^b^ ± 0.01

Results are expressed as mean ± SD. AIN-93 M diet: negative control group (n = 10/group); cafeteria diet (CAF): positive control group (n = 10/group); and caferia diet with whole soybean flour (CAFS) (n = 10/group). To compare the experimental groups Tukey test was used. For each evaluated characteristic means followed by the same letter in the line are not significantly different at 5% probability.

The dietary intake was evaluated by recording the daily food intake, and weight gain was obtained from the difference between the initial and final weights of the animals. The Feed Efficiency Ratio (FER) was determined from the ratio of the weight gain of the animal and the consumption of the experimental diet.

At the end of the experiment, the animals were fasted for 12 hours, anesthetized with ether and subjected to euthanasia by exsanguination. Their blood was collected and centrifuged at 1000 × g for 15 minutes to obtain the serum that was stored at -20°C, for biochemical serum samples, and -80°C for peroxidation analysis. The following organs were removed: the liver, proximal (duodenum) and distal (ileum) small intestine, kidney, lung and testicles. The liver, kidney, lung and testicles were kept in liquid nitrogen and lyophilized for subsequent biochemical analyses. Samples of the liver and portions of the small intestine, duodenum and ileum were fixed in Bouin's fluid for histological studies.

The feces were collected during the last week of the experiment and were subjected to drying and grinding for determination of the water and lipid content, according to the methodology of AOAC [52].

It must be mentioned that this experiment was approved by the Ethics Committee on Animal Research of the Federal University of Minas Gerais (UFMG-CETEA), Case 212/2009, and was conducted in accordance with the Ethical Principles of Animal Experimentation (CETEA / UFMG).

### Serum parameters

The total cholesterol, HDL fraction and triglycerides were determined using the enzymatic colorimetric method; glycated hemoglobin (Hb1Ac) was determined by the ion exchange method; while the activity of the aminotransferases was checked using the UV kinetic method. All analyses were performed using the commercial kits (Human do Brasil®), in accordance with manufacturer guidelines.

### Lipid peroxidation analysis

Lipid peroxidation was estimated in the serum and tissue homogenates of the lyophilized liver, lung, kidney and testicles through the Thiobarbituric Acid Reactive Substances (TBARS) test, according to the methodology described by Buege and Aust [53]. In order to obtain the homogenates, tissue lyophilisates were resuspended in a 0.1 M phosphate buffer, pH 7.4, at 1:10 (m / v).

### Hepatosomatic index (HSI)

After excision, the animal's liver weights were registered and used to calculate the hepatosomatic index, which represented the relationship between the organ weight and the weight of the live animal, after undergoing a 12-hour fast.

### Histological analysis

The fragments of the liver, duodenum and ileum were collected from six animals in each experimental group. The intestine and liver portions were fixed in Bouin's fluid for a minimum of 24 hours and preserved in 70% alcohol. Nine semi-serial transversal sections, 3 μm in thickness, using hematoxylin/eosin stain were obtained from the duodenum, ileum and liver. The slides were analyzed under the light microscope Olympus CX31, and the images were obtained with a digital camera SC 020, through the GetIt Analysis software, Olympus. In order to measure the villous height, crypt depth and thickness of the internal and external muscle layers, we used the Image-Pro Plus® version 4.5 software. The images captured were utilized for visualization and quantification of the fat regions of the liver tissue; the morphometry of the circular and longitudinal muscle layers of the intestine was done by using the 10× objective, whereas the morphometry of the villi and crypts in the duodenum and the ileum was used visualized using a 4× lens.

**Figure 1:**
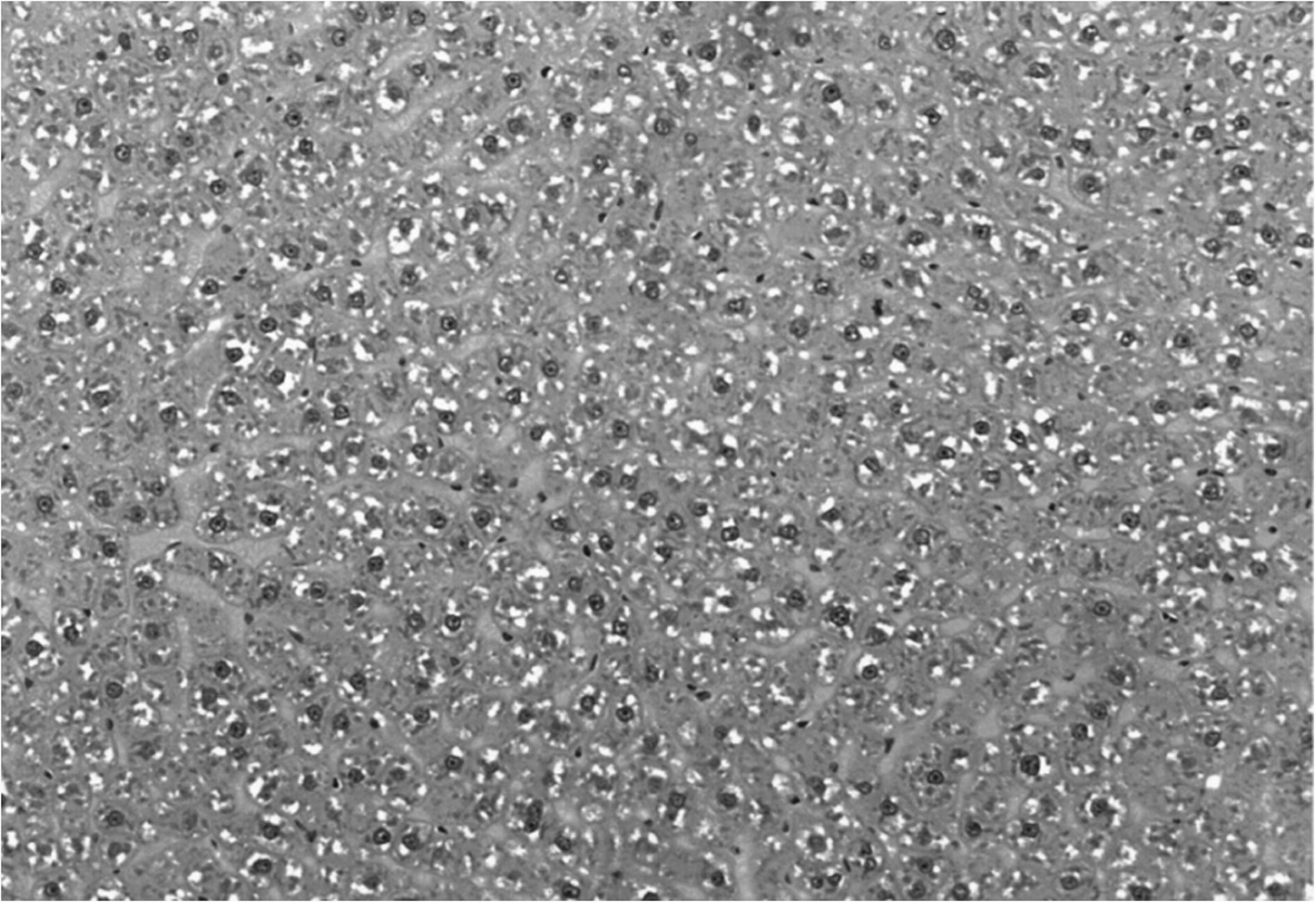
**Photomicrography of the liver of animals fed with cafeteria diet.** Bar: 3 μm. Hematoxylin-Eosin staining.

The fat deposition in the liver was determined using the computational quantification of fat droplets and the values were obtained by applying the algorithm developed programming language Open Source SciLab, version 4.1 (INRIA, ENPC, 2006), utilizing the principle of thresholding (Figure 1) [54].

**Figure 2:**
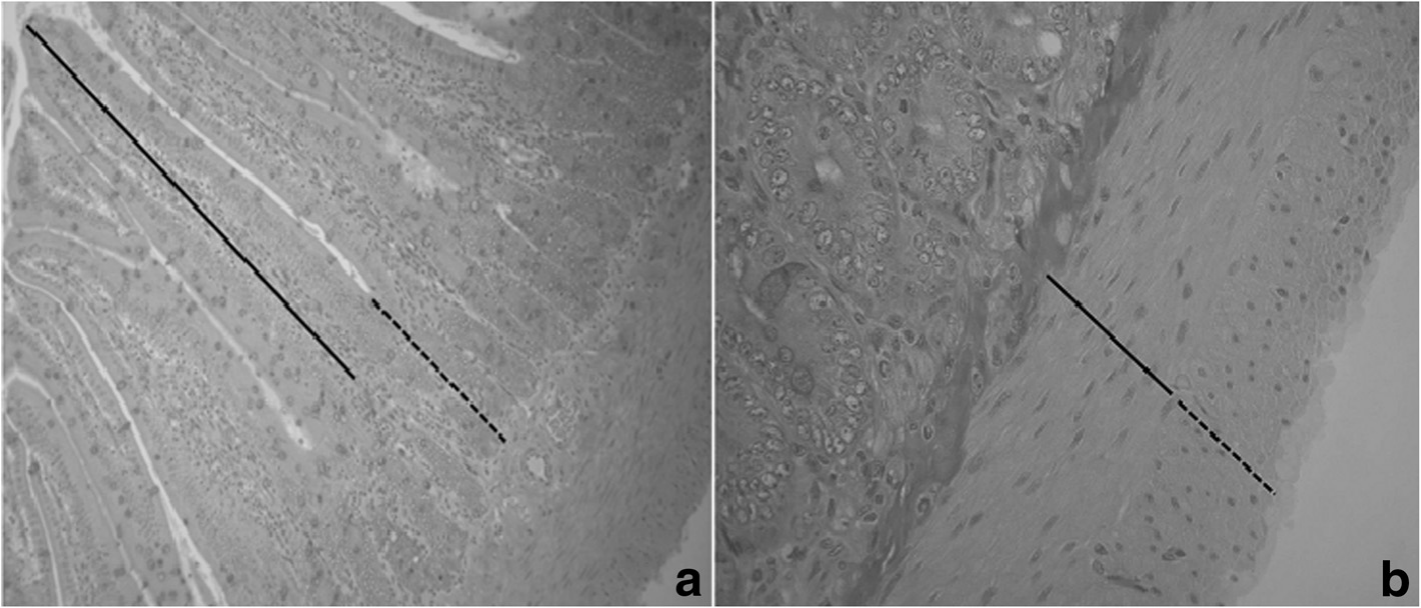
**Graphical representation of the measurements in the intestine of animals. 2a**: 40×; **2b**: 100×.

In the two portions of the intestine, the measurements of villous height, crypt depth and the thickness of the circular and longitudinal muscle layers were obtained using the Image Pro-Plus® version 4.5 software (Media Cybernetics) [55] (Figure 2). To take these measurements, 10 random fields were selected per animal, totaling to 40 measurements for each parameter analyzed.

### Estatistical analysis

The experimental design was in blocks, with three treatments and ten replications. In order to proceed with the histological analysis of the intestine, four replications were used. The data of weight gain, feed intake, biochemical parameters, water, lipid and fecal contents, hepatosomatic index, lipid peroxidation and intestinal histomorphometry were analyzed using ANOVA and the results expressed as mean ± standard deviation. Significant differences between the groups were detected by the Duncan test, using the Statistical Analysis Software System for (SAEG) version 9.1 [56], licensed by the Federal University of Viçosa. The Kruskal-Wallis test was used to analyze the hepatic fat deposition, using the Sigma STAT software version 2.03. The level of significance was set at 5%.

## Abbreviations

ALT: Enzyme alanine aminotransferase; AST: Enzyme aspartate aminotransferase; CAF: Cafeteria diet; CAFS: Cafeteria diet with soy flour; CD: Crypt depth; CVD: Cardiovascular disease; DI: Dietary intake; FER: Feed efficiency ratio; HbA1c: Glycated hemoglobin; HIS: Hepatosomatic index; TBARS: Test thiobarbituric acid reactive substances; TCML: Thicknesses of the circular muscle layer; TLML: Thicknesses of the longitudinal muscle layer; VH: Villous height; VLDL: Very low density lipoprotein.

## Competing interest

The authors declare that they have no competing interest.

## Authors’ contributions

GFA, CGA, MISD, SMRR and HSDM contributed the design and conducted the study, collection, analysis, interpretation of data and writing of the manuscript. ACRE contributed the design and conducted the study, collection and analysis of the data. LAB contributed to the study, analysis and interpretation of the data. All authors read and approved the final manuscript.
